# Declaring Racism a Public Health Crisis in the United States: Cure, Poison, or Both?

**DOI:** 10.3389/fpubh.2021.676784

**Published:** 2021-06-18

**Authors:** Lilliann Paine, Patanjali de la Rocha, Antonia P. Eyssallenne, Courtni Alexis Andrews, Leanne Loo, Camara Phyllis Jones, Anne Marie Collins, Michelle Morse

**Affiliations:** ^1^National Birth Equity Collaborative, Washington, DC, United States; ^2^National Birth Equity Collaborative, New Orleans, LO, United States; ^3^EqualHealth's Global Campaign Against Racism, Brookline, MA, United States; ^4^Department of Global Health, University of Washington, Seattle, WA, United States; ^5^UCLA Center for the Study of Racism, Social Justice, and Health, UCLA and Charles Drew University Covid-19 Equity Taskforce, Los Angeles, CA, United States; ^6^Cityblock Health, Brooklyn, NY, United States; ^7^Department of Anthropology, Tufts University, Medford, MA, United States; ^8^Rollins School of Public Health at Emory University, Atlanta, GA, United States; ^9^Morehouse School of Medicine, Atlanta, GA, United States

**Keywords:** structural racism, health equity, social justice policy, accountability, public health crisis, pharmakon, black lives matter, decolonial practice

## Abstract

Declaring racism a public health crisis has the potential to shepherd meaningful anti-racism policy forward and bridge long standing divisions between policy-makers, community organizers, healers, and public health practitioners. At their best, the declarations are a first step to address long standing inaction in the face of need. At their worst, the declarations poison or sedate grassroots momentum toward anti-racism structural change by delivering politicians unearned publicity and slowing progress on health equity. Declaring racism as a public health crisis is a tool that must be used with clarity and caution in order to maximize impact. Key to holding public institutions accountable for creating declarations is the direct involvement of Black and Indigenous People of Color (BIPOC) led groups and organizers. Sharing power, centering their voices and working in tandem, these collaborations ensure that declarations push for change from the lens of those most impacted and authentically engage with the demands of communities and their legacies. Superficial diversity and inclusion efforts that bring BIPOC people and organizers into the conversation and then fail to implement their ideas repeat historical patterns of harm, stall momentum for structural change at best, and poison the strategy at worst. In this paper we will examine three declarations in the United States and analyze them utilizing evaluative criteria aligned with health equity and anti-racism practices. Finally, we offer recommendations to inform anti-racist public health work for meaningful systematic change toward decentralization and empowerment of communities in their health futures.

## Background

“*By itself, however, naming racism does not ensure equity. We must also tackle the underlying mechanisms by which white supremacy and structural racism preserve themselves. Otherwise, naming racism will serve as a substitute for actually eradicating it.”*

*-Dr. Chandra Ford, in response to the CDC declaration of racism as a public health threat* *(**1**)*

The World Health Organization's (WHO) Conceptual Social Determinants of Health (SDoH) framework (2) demonstrates how social, economic, and political factors such as income, education, occupation, gender, race, and ethnicity influence a person's socioeconomic position that, in turn, plays a role in determining health outcomes. Racism and discrimination are also key determinants of health (see Table 1).

**Table 1:** Definitions.

**Race and Racism**
Race is a socially and politically constructed way of grouping people (3 ). Racism is a system of structuring opportunity and assigning value based on how one looks, which unfairly advantages some, disadvantages others, and saps the strength of the whole society (4 ). Historically, racism has operated as a socio-political and economic construct rooted in the violation of dignity, humanity, personhood, and self-determination of communities and their access to land, resources, and basic human rights. Racism operates across ecological levels and societal structures (4 ). Utilizing Dr. Jones' theoretical framework, this paper identifies racism operating at three levels: internalized, interpersonal, and structural. Internalized racism occurs when members of racialized groups accept negative messages about their abilities and intrinsic worth. Interpersonal racism manifests as prejudice and discrimination through differential actions toward racialized groups. Institutional or structural racism is differential access to the goods, services, and opportunities of society by race which can manifest as inaction in the face of need.
**Crisis, Emergency, Issue**
A public health issue is one that affects a significant portion of a specific population. According to the WHO, an emergency describes “a state that demands to be declared or imposed by somebody in authority, who, at a certain moment, will also lift it.” It is usually defined in time and space and requires threshold values to be recognized; it also implies rules of engagement, an exit strategy, and mobilization of resources/budget (5). In contrast, a crisis implies the possibility of an insidious process that cannot be defined in time, and that can exhibit different layers/levels of intensity. A crisis “may not be evident, and it demands analysis to be recognized” (5). Conceptually, “crisis” can cover both preparedness and response. Therefore, the term crisis is the most appropriate term to use when it comes to addressing and defining the state of structural racism and its impacts on health. The threshold values and limits of the term “emergency” are insufficient and the term “issue” too vague.
**White Supremacy Culture**
While “white supremacy” often conjures images of individual or group acts of overt and racially motivated acts of violence, “white supremacy culture” address normalized and pervasive daily and subtle manifestations that support the maintenance of a white supremacist system. White supremacy is a system in which those who benefit from whiteness maintain power and privilege through the oppression and exploitation of those who are not included in the definition of whiteness, with Black and Indigenous People of Color (BIPOC). Culture is the behaviors, beliefs, values, and symbols that they accept, generally without thinking about them, and that are passed along by communication and imitation from one generation to the next. Cultural racism is how the dominant culture is founded upon and then shapes the society's norms, values, beliefs, and standards to validate and advantage white people while oppressing BIPOC (6). White supremacy culture maintains that the cultural behaviors, beliefs, values, and symbols of white people is superior to that of people of color. As identified in *Dismantling Racism: A Workbook for Social Change Groups*, this includes perfectionism, worship of the written word, sense of urgency, defensiveness, quantity over quality, only one right way, paternalism, either/or thinking, power hoarding, fear of open conflict, individualism, progress is bigger/more, objectivity, and right to comfort (6).

The American Public Health Association's (APHA) 2016 Presidential Initiative, a National Campaign Against Racism (7) asserts that racism is:

- a social system with multiple dimensions: individual racism is internalized or interpersonal; and systemic racism is institutional or structural, and
- a system of structuring opportunity and assigning value based on the social interpretation of how one looks, that
- unfairly disadvantages some individuals and communities,
- unfairly advantages other individuals and communities, and saps the strength of the whole society through the waste of human resources.

When examining the impact of a resolution or a declaration of racism as a public health crisis, it helps to take a heuristic approach while utilizing a pharmakon framework. A heuristic approach is an accurate, reliable and generalizable way to examine a concept for problem solving or learning how to problem solve (8). When we examine the resolution or declaration as a remedy for structural racism, we must consider the potential for harm or poison (pharmakon), that can occur when that solution (declaration of racism as a public health crisis) stalls momentum for meaningful material anti-racism policy and program implementation. However, we have to be vigilant and thoughtful of the ways this can be implemented because performative actions, missteps and intentions can be counterproductive.

The concept of performative allyship in anti-racism is understood as someone from a non-marginalized group professing support and solidarity with a marginalized group, but in a way that is not helpful and potentially even harmful (9). This work examines not simply the performative nature that declarations can embody, but also the implications of non-performativity. The non-performativity of anti-racism within institutional speech acts has been described by Sara Ahmed as holding the intent to do nothing. Institutional anti-racism speech is not a failed performance that did not reach its intended audience, but rather is intended to be non-performative. When, “the failure of the speech act to do what it says is not a failure of intent or even circumstance, but is actually what the speech act is doing,” therein lies the potential for harm in a speech act which aims to cure (10).

According to the philosopher Achille Mbembe in his book *Necropolitics*, the ultimate expression of sovereignty resides in the power and capacity to dictate who lives and who dies and draws on the concept of pharmakon as “a medication that acts at once as remedy and as poison” (11). Derrida defines “pharmakon” as the desire to bring healing while being blind to harm (12). It is within this framework that we analyze the declarations of racism as a public health crisis while we consider the potential unintended consequences. This suggests that a resolution or declaration can simultaneously be the remedy and the poison. Guided by the values of health equity, we will build on the concept of pharmakon to propose paths forward to magnify and expand the impact of such declarations in addressing the systemic oppression and violence which leads to racialized health inequities. Within the lineage of Derrida and Foucault, Mbmbe's conceptualization of biopower and biopolitics depart from the Eurocentric view to include a framework of power with a critical lens of colonialism, post-coloniality, and conceptualizations of aliveness within a framework of the “living dead” whose bodily sovereignty are controlled by racialized state politic (11). That is, this paper aims to address remedies to the potential implications of state-sanctioned death through non-performativity in declarations of racism as a public health crisis. If the data is disaggregated, the mortality disparities are clear, if racism has been declared a crisis, and still no meaningful action is taken, then these declarations become one more way that “contemporary forms of subjugation of life [give] to the power of death” (11).

Catalyzed by the murder of George Floyd on May 25th, 2020 by a police officer in Hennepin County, Minneapolis, over 180 declarations have been made across public health institutions, US counties, cities, and states in <1 year (13). Though declarations are not uniform in their scope, most have sought to raise awareness about how structural racism and systemic inequities negatively impact population health. This praxis is aligned with the three principles for achieving equity: (1) Valuing all populations evenly and equally more broadly; (2) Evidence-based approaches to recognize and rectify historical injustices; (3) Providing resources according to need. Using these steps as a guide, governments have their best chance to conquer health inequities through meaningful implementation of declarations (14).

## Historical Context

### Legacies of Racism in Public Health

The history of public health is a complicated story of science and colonial, post-slavery social systems where good faith policy narratives implicitly and/or explicitly put into practice violent systems of restrictive health access that perpetuate inequity (15). While the role of anti-racism declarations in impacting health outcomes, addressing the social determinants of equity (SDoE), and decreasing inequities is still being conceptualized and actualized in measurable, concrete, and consistent ways, there is historical precedent.

Within the context of necropolitical biopower and colonialism, it is important to name the ongoing struggles of Indigenous sovereignty globally. Within the United States, the historical legacies of land theft, genocide, ongoing violation of the over 500 treaty rights that were signed between 1778 and 1781 (16), and continued erasure of Native American, Alaska Native, Hawaiian, and other Indigenous communities contribute to the lack of sovereignty and disparate health outcomes seen today (17), particularly during the COVID-19 pandemic (18). An example of non-performativity within public health is continued genocide through data erasure and misclassification (19). Lack of data directly impacts the ability of local, state, federal and tribal public health authorities to address the COVID-19 virus and limits policy makers' ability to make data-driven decisions for equitable policy and resource allocation (20). Few, if any declarations, acknowledge or address Indigenous populations within declarations. Within a context of Indigenous erasure, non-performativity is made visible by its absence.

Since World War II, the creation of international governing bodies has increasingly been used to affirm state legitimacy, particularly through the ratification of human rights principles and treaties. Notwithstanding their intended effects, these accords have led to an increase in abuse (21, 22). Known as “radical decoupling,” this phenomenon reflects the harms of disconnection between practice and policy and provides insight to the pharmakon phenomenology (23), Human Rights as Myth and Ceremony found that while signing declarations was associated with improved human rights practices, it did not necessarily translate into improved human rights outcomes (24). Meir et al. wrote that accountability; non-discrimination and equality; and community participation are “crucial to realizing all human rights” (25). Our collective process of transmuting the poisons of structural racism into solutions for structural racism requires not only commitment to specific protocol, but careful practice and work (26, 27).

Black Lives Matter (BLM) has created a window of opportunity for anti-racism dialogue and action in health sectors and beyond (28). Catalyzed by the acquittal of George Zimmerman for the murder of Trayvon Martin, the courage of this movement elevated the conversations of the countless unjust murders and deaths at the hands of police from the living rooms of the Black community to a global dialogue on state-sponsored violence against people of color in all spaces (29). These conversations were reignited during the COVID-19 pandemic with the murder of Mr. George Floyd and the revelation of structural gaslighting not only within policing in America but also within the medical system surrounding his autopsy (30). The recent death of Dr. Susan Moore from complications of COVID-19 further exemplifies how the systems and policies in the US across all public and private sectors are not set up for safety for people of color and rather than take responsibility, often blame them for poor outcomes (31). Her heartbreaking video referencing the inequitable care that she received *as a doctor* was punctuated by words that Black and Indigenous People of Color (BIPOC) feel around the world “I put forth and I maintain, if I was white I wouldn't have to go through that” (32).

Thankfully, the public health community has taken the lead in calling attention to how racism is operating in public health and medical spaces (33), how health workers and healthcare institutions have been complicit with structural racism (34), and how critical race theory can help us to better understand and challenge the impact of racism on health and well-being (35). Trainees across the health professions have responded by organizing protests, founding new organizations like White Coats for Black Lives (36), and pushing for institutional change such as ending race correction of kidney function (37). This is evident by the actions of the American Medical Association (AMA) declaring racism a public health threat and the removal of the public display of the founder due to their racist past, along with the removal of their name off of one the most prestigious annual awards (38, 39). The rising tide of declarations of racism as a public health crisis is an opportunity to not only drive this momentum forward, but to transform it from mere anti-racism declaration to anti-racism action through meaningful processes of accountability rooted in health equity principles and decolonial practices.

### Declaration Origin Story: WPHA

The Wisconsin Public Health Association (WPHA) is the largest and most recognized membership association for public health professionals in Wisconsin. Catalyzed by the reality of injustice in local maternal/child health, the events in Charlottesville, Virginia in 2017, and the 2016 launch of a National Campaign Against Racism by the American Public Health Association (7, 33), At-Large Representative for the Board of Directors Lilliann Paine, MPH created a Racial Equity Workgroup for the Wisconsin Public Health Association (WPHA). Driven to get racial equity on the strategic planning agenda of the WPHA, Paine along with colleagues Jessica LeClair and Colleen Moran led the development of the Racial Equity Workgroup. This trio emphasized the need for WPHA to create educational opportunities and build capacity (e.g., board orientation, conference, external evaluator, trained facilitator) to have deliberate dialogue regarding whiteness, power, and privilege. It was the desire of these founding members to establish racial equity as a core element in the WPHA.

A conversation about feeling safe and included at the May 2017 WPHA annual conference sparked a journey that resulted in the creation of the WPHA Racial Equity Workgroup. During the strategic planning process, the Board discussed proactive steps to assess policies, processes, and social relationships that contribute to the exclusion of racialized communities.

The Board approved the Racial Equity Workgroup in September 2017. An invitation was extended to the membership for potential committee members. Eleven applicants were selected based on their experience working toward racial equity and their interest in serving on the Racial Equity Workgroup. The Workgroup's charge was to recommend activities to support public health professionals in their work to address racial inequity in public health.

In August of 2017, the WPHA Board discussed the events in Charlottesville and events following that tragedy. A written statement asserted that WPHA continues to support APHA's policies on race and hate crimes (40). The Board emphasized the desire to continue this important conversation and identify potential strategies to address these issues through their current and future work. One immediate opportunity was to expand the WPHA 2010 resolution titled *Achieving Health Equity* to include racism among other inequities for the WPHA Annual Meeting in May 2018 (40).

In December 2017, the Workgroup located resources, drafted a workplan, using the APHA 2016 Presidential Initiative as a model to prepare a resolution naming racism as a public health crisis. Initial resistance grew out of discomfort about making the implicit explicit and pointing out biases around dominant culture and the minimization of differences. WPHA members approved the resolution at the May 22, 2018 Annual Business Meeting. This was the blueprint and catalyst for recognizing the need for shared responsibility of exercising racial equity to achieve the organization's mission. This was the remedy.

One of the strong considerations for the WPHA 2018 Resolution was to conduct an organizational assessment of internal policy and procedures using the Beloved Community Survey to ensure racial equity is a core element for the organization. The survey results assessed on a scale of nine specific domains: embracing conflict, seeking reconciliation, seeing, redeeming qualities, forgiving, loving, moving toward liberation, balancing, being radically open, and struggling with fear. The findings of the survey did not translate into improved outcomes but rather created conditions where Beloved Community could become a practice (41).

## Evaluative Criteria

As the WPHA story illustrates, it is not enough to make a declaration of racism a public health crisis. Public health has to be in alignment with the radical imagination and with the communities it serves, works to protect, and center. Accountability with a material commitment toward first naming and then reckoning with lack of racial consciousness, protection of privilege, and internalized oppression and dominance. The desired change would be a raised awareness and critical analysis of racial consciousness and the articulation of power and difference at a structural level.

Upon reviewing the literature, policy and financial documents, legal codes, grassroots mandates, the work of community-based health organizations, and conducting stakeholder interviews, we identified evaluative criteria to assess if declarations integrated meaningful accountability measures. The three declarations highlighted below were chosen based on their unique approach to the integration of principles of racial equity and their development pre or post the killing of George Floyd in order to demonstrate the evolution of the trend toward making declarations. Each declaration implemented different strategies: working inside-out (Milwaukee County), coalition building (Ventura County), and community organizing (King County).

Declarations were evaluated according to whether they are: a*ctionable*, f*inancially responsible to communities most impacted, address the structural determinants of equity/inequity*, and include meaningful community *participation* (see Table 2).

**Table 2:** Evaluative criteria for accountability in addressing racism as a public health crisis.

**Criteria**	**Definitions**
Actionable	Declarations can propose a theory of change to public health institutions toward achieving a collective vision of racial equity in health. Plans can drive institutional and structural change if they are actionable (42). This requires resources to implement time, money, skills, and effort. It requires local governments' will and expertise to change policies, practices, habits, and cultures (43). For example, declaring a national holiday in recognition of children descended from slaves does little to repair inequity in education. In contrast, eliminating school suspensions and implementing restorative justice practices serves to disrupt the school to prison pipeline (44).
Financially responsible	Cost effectiveness, while a traditional consideration in policy analysis is distinct from financial responsibility (45). Financial responsibility uses an equity lens in particular highlighting Dr. Jones' third equity domain providing resources according to need (4). It considers intangible individual, community, and societal costs and additionally considers how funding is allocated and creates space for equitable funding analysis, tools, and concepts of reparations and debt forgiveness (46). This can include use of race equity budget tool, reparations frameworks, or debt forgiveness.
Addresses structural determinants of equity	A structural determinant of equity (SDoE) approach will frame any interventions, models or programs in focusing on structural or root causes. For example, as defined by Healthy People 2020 and Healthy People 2030, structural determinants of health (SDoH) are “the conditions in the environments where people are born, live, learn, work, play, worship and age that affect a wide range of health, functioning, and quality-of-life outcomes risk” (47). Understanding health disparities requires a holistic perspective where “social determinants are often the root causes of illnesses, disease and disparities” because of racialized, systematic oppressions imposed and perpetuated (48). SDoE challenge the current distribution of SDoH by addressing factors that determine the range of contexts observed in each place and time and the distribution of populations in those contexts (49). SDoE focuses on structural or root causes, recognizing that addressing health disparities requires a holistic perspective; authentic processes in mitigating inequity require addressing the unequal allocation of power and resources (50). This includes disaggregation and stratification of data by race (51).
Participatory	Meaningful engagement of community across all levels of public health practice through culturally grounded and community led processes, programs, and interventions (52) through participatory frameworks allow for analyses of power, profit, control, exploitation, ableism, oppression, violence, and trauma across health systems with a focus on addressing harm and generational healing (53). Participatory processes can serve as an aspect of healing justice if it seeks to address systemic oppression through community/survivor-led responses to support community well-being across ecological levels and ecosystems (54). Participatory engagement and processes includes, but is not limited to, building and maintaining relationships to the land and nature, centering patient autonomy and consent, and foregrounding sustainability as a political practice (the creation of advisory councils, participatory budgeting processes, engagement in the policy process, etc.) (55).

## Milwaukee County, Wisconsin: Inside Out Strategy

### Description

Inspired by the WPHA unanimously passing a resolution that declared racism a public health crisis the year before, Milwaukee County Executive Chris Abele signed the first public declaration, a rectified version of the WPHA's resolution, on May 20, 2019. The Common Council for the City of Milwaukee followed suit on July 30, 2019 (56). There are 19 cities in Milwaukee County, with a population of around 950,000. Prior to the murder of Mr. George Floyd, only eight jurisdictions (cities or counties) in the country had declared racism a public health crisis. Milwaukee County was the first.

The Racial Equity Assessment Report (Beloved Community Survey) was commissioned by WPHA through a request for proposal process identified by the Ubuntu Research and Evaluation. Milwaukee County did not conduct an environmental scan or internal assessment. It was important that the third-party organization be led by a woman or BIPOC team, along with having the lived experience of communities that are historically oppressed and traditionally marginalized. The findings of this report created the framework and context for policy changes that officials could use to execute on the commitments outlined in the declaration (41). Informed by the WPHA resolution, the Milwaukee County Executive Board took steps to create the first municipal ordinance in the country to make a formal declaration where race is acknowledged as a social construct, to assess internal policies and procedures using race equity as a core competency, and to advocate for policies to improve health in communities of color. In addition to concrete changes in policy language, representation in staffing and community investments, and utilizing a race equity budgeting tool across all departments, this upgraded view of health helped to disaggregate COVID-19 data by race at the beginning of the COVID-19 pandemic shedding light on its disproportionate impact on Black Americans. The City of Milwaukee followed a continuum on becoming an anti-racist multicultural organization (57).

### Analysis

#### Actionable

The initial declaration by the WPHA was a resolution which politically holds more symbolic than actionable significance. However, in May 2020, the Municipal County created an ordinance, Chapter 108 of the municipal code: “Achieving Racial Equity in Health.” Transforming this resolution into an ordinance created the legislative teeth and accountability for the objectives described in the declaration. Since its signing, Milwaukee county has provided 11,000 training hours to over 3,000 employees (about 75% of their total staff). They became a member of the Government Alliance on Race and Equity (GARE), who helped them develop training and toolkits. They were among the first locales to report racial disparities in the COVID-19 pandemic, which is one of the more compelling outcomes despite not being an original goal of the declaration (58).

#### Financially Responsible

Through the partnership with GARE, they developed a racial equity budget tool. Each department went through a budget request using this race equity lens to see burden and benefit in 2020 to determine the 2021 budget priorities (59). This budget tool aligns with four strategic objectives: diverse and inclusive workforce, people-focused design, employee perspective, and equitable practice.

#### Addresses SDoE

Milwaukee is made up of 19 municipalities and each one has its own health department except for Milwaukee County. When the County declared the COVID-19 public health emergency on March 13, 2020, the City of Milwaukee Health Department led the pandemic response.

Due to the foundational work done at both the County and City level the year before and utilizing WPHA's 2018 resolution as a blueprint to declare racism a public health crisis, the City of Milwaukee Health Department was able to document and address racial disparities during COVID-19. This work would not have been possible without the actions that flowed from the preexisting declaration of racism as a public health crisis. Moreover, they were the first to report race stratified COVID-19 data in the country (58). This enabled them to allocate $77 million dollars for mortgage assistance, eviction prevention, housing, and funding to community-based organizations and invest in equity across the county.

#### Participatory

Since the Declaration, Milwaukee county has created a health and race equity framework to analyze policies, practices, and procedures rooted in white supremacy culture and power. This process has led to the first strategic plan in 20 years with 3 identified focus areas aligned with power mapping strategies. These focus areas center engagement and participation of communities most impacted by structural racism in both internal and external processes: (1) Create Intentional Inclusion; (2) Bridge the Gap in Addressing Disparities; (3) Invest in Equity.

## Ventura County, California: Coalition Advocacy

### Description

Ventura County's Declaration (60) is framed as “the first step of many against the institution of racism” following the murder of Mr. George Floyd. According to Black Lawyers of Ventura County (61) the resolution was drafted after they released a statement, which was modeled after the 8 Can't Wait policy recommendations. Eight Can't Wait was a social media campaign that demanded immediate actions to reduce police violence (62).

The final Ventura County resolution, which was drafted in collaboration with BLVC, focused on Ventura County law enforcement agencies based on the recognition that Black people are under existential threat due to burgeoning police killings. It recognizes how the school-to-prison pipeline ties to the incarceration of BIPOC youth and specifically calls out their policy to not use law enforcement for disciplinary practice. In addition, a diversity and inclusion officer was hired within Ventura County Public Health.

According to Ventura County Public Health and members of the BLVC, Ventura County law enforcement agencies have implemented six of the eight actions with many of the policing reforms put into effect prior to Ventura County's resolution.

### Analysis

#### Actionable

The declaration exists currently as a resolution and is framed as a pledge rather than a deliverable set of objectives. It has set the stage for community forums, employee training, and collaborative conversations with community-based organizations to surface specific concerns in the hopes of problem solving in the future (63). As it stands, it runs the risk of not being actionable as there are no explicit deliverables articulated.

The 8 Can't Wait Campaign has been criticized (62) for its reliance on faulty data science and statistical analysis. The campaign argues that if eight policy shifts are made at the city level, it can reduce police killings by 72 percent (64). “But the data and study design do not support that staggering statistic put forth in the least bit,” activists Cherrell Brown and Philip V. McHarris said in a statement published on June 5, 2020 (65). It has been criticized by activists as irresponsible and creating an out for leaders and politicians looking for alternatives to more transformative abolitionist demands (66).

#### Financially Responsible

While the resolution resolves to invest in economic initiatives that support housing, business, and education and pledges to expand partnerships with public health institutions and non-profit organizations involving social workers and mental health professionals, it does not have any budgetary impact. It is unclear with the resources available at this writing whether there is a shift of resources to keep initiatives budget neutral while investing in the priorities outlined in the declaration.

#### Addresses SDoE

While it is impossible to understate the legacies of harm that anti-Blackness and white supremacy have caused to African American populations in the US, it is equally important for counties and health institutions to consider the additional ways that racism is operating in their context. Of the 1,300 police shootings in California (67) since 2013, 20 occurred in Ventura County (68), amounting to ~2.2 shootings per year.

There is little available data on racial disparities in SDoE in Ventura County; the California Health Places Index (69) from the Office of Health Equity disaggregates by several identified determinants of health, but does not disaggregate by race. Current California standards and tracking using place-based indicators systematically underrepresent Pacific Islander and Native American populations (70). A 2015 article indicates that immigrant Indigenous communities in Ventura County reported discrimination, insufficient employment opportunities, access to food and housing, and lack of transportation (70). This same study found that many adults in the community were able to secure healthcare for their children but not for themselves.

While the declaration emphasizes the role of eliminating discriminatory policing as a determinant of health, addressing anti-Black racism, and legacies of slavery cannot be fully realized if they are achieved by perpetuating Indigenous erasure and erasure of other communities of color. Addressing over policing of Black communities without addressing SDoE for such a large immigrant, undocumented, and non-English speaking population, Ventura County runs the risk of pharmakon “desire to bring healing while being blind to harm.”

#### Participatory

The document proposes to establish a working group of experts to study the delivery of healthcare to underserved populations, and to continue to incorporate diversity and equity training for county employees. The final draft included input from the NAACP, Black Lawyers of Ventura County, local law enforcement agencies, public health experts, the district attorney's office, and Ventura County Human Resources (71).

There was a missed opportunity in addressing the full realm of participatory praxis by excluding broader community participation, particularly the Indigenous and Hispanic communities that make up the majority of the minority population in the county.

## King County, Washington: Community Organizing

### Description

On June 8th, 2020, Black-led movements and protests following the murder of Mr. George Floyd were successful in occupying a police precinct in a neighborhood in Seattle after a week of nightly confrontation with police who excessively used teargas. This area became known as the Capitol Hill Organized Protest (CHOP) and was a community hub and mutual aid center with free food distribution, community housing, medical teams, daily art and political education classes, and a community garden (72). While CHOP was cleared by police in July 2020, there were daily and nightly marches for nearly 300 days since the murder of Mr. George Floyd. CHOP Organizers had three main demands: cut Seattle's $409 million police budget by 50 percent, shift funding to community programs and services in historically Black communities, and ensure that protesters would not be charged with crimes. Seattle has cut the police budget by 5% and those funds are being reallocated utilizing a participatory budgeting process involving community leaders.

At the same time CHOP was being formed, local organizers and faculty at the University of Washington wrote an open letter to public health officials on June 6th, which garnered over 2,000 signatures and included demands to declare racism and policing public health emergencies and to defund the police to reallocate resources (73). They also created a policy around decriminalizing the University of Washington campus (74). Over 10,000 healthcare workers marched on June 10, 2020 and held a rally outside the Mayor's office demanding action (75).

The next day, on June 11, 2020 King County declared racism a public health crisis (76). The same executive who made this declaration had also created a roadmap to Zero Youth Detention in 2017, while building a $210 million dollar new youth jail in a historically Black and immigrant neighborhood (77, 78).

### Analysis

#### Actionable

Nearly 300 organizations and over 43,000 people have signed on to decrease the police budget by 50% resulting in a step in the right direction with a 5% cut. While much effort has been placed to defund and reallocate policing budgets, King County Public Health has also created other mechanisms within public health, including a community engagement compensation fund, building new models to serve residents in community safety, redesign fare enforcement on public transit, increase translation access for public health materials, and expand community engagement in budget development for fiscal years 2023–24.

#### Financially Responsible

In 2021, King County has budgeted $400 million in securing permanent housing, nearly double the last budget (79). However, it is unclear who this housing supports. In the previous budget, the approach to priorities were strengthening financial practice and improving operations. For this biennial budget, the priorities were replaced with anti-racism, criminal legal reform, and community involvement to inform their approach (79).

As part of an eight-point anti-racist budgeting priority, they plan to invest $6.2 million in “Restorative Community Pathways,” invest $750,000 to co-create and implement alternatives to policing in urban unincorporated King County, divest $1.9 million in detention by continuing limits on jail population, and invest in community engagement.

#### Addresses SDoE

Under the leadership of County Executive Ron Sims, in September 2020, King County Public Health (KCPH) conducted an Equity Impact Review through their Equity and Social Justice Initiative, which was founded in 2008 (80). Despite this commitment to advancing equity (81), communities of color still experience disproportionate risk to COVID-19 (82), as well as access to care. There are over 30 identified immigrant populations and 20 official languages spoken in King County.

Due to existing equity protocol as well as emergent community consultant programming, King County Public Health allocated $60 million for acquisition and development of isolation and quarantine facilities, PPE, and shelter de-intensification in neighborhoods most impacted by COVID-19 (79). This program was successful due to the meaningful engagement of the community through their community navigator COVID-19 response program and advisory group.

However, even prior to the pandemic, the gap in life expectancy was as high as 18 years in conditions such as ischemic heart disease and drug use disorders between immigrant and BIPOC neighborhoods and white neighborhoods (83). These facts underline the importance of pairing declarations with deliverables.

#### Participatory

As an initial pandemic response, King County created a Pandemic and Racism Community Advisory Group (84) as well as a team of cultural and faith based navigators to lead equity responses to the dozens of immigrant, minority, and religious communities residing in King County.

King County Equity Now (85) and Decriminalize Seattle (86), two Black-led movement-based organizations, are coordinating a participatory budgeting process for the “Public Safety” parts of the 2021 city budget. Community members will develop a budget proposal for the dollars currently spent on the Seattle Police Department, Municipal Court, and the City Attorney's office (the city prosecutors). They have created a team of community-based researchers across demographic areas and from communities impacted by the carceral system and racialized violence to determine how to reallocate $30 million dollars out of policing and into community alternatives (87).

## Recommendations

These declarations demonstrate the importance of not only acknowledging historical injustices, but also clearly defining steps toward dismantling structural harm. Press releases from mayors, statements by health boards, newly formed racial equity positions and centers, and commitments to policy changes should be accompanied by concrete action plans with deliverables that address and confront several systemic and structural issues, which include but are not limited to police brutality, inequitable education, housing segregation, environmental degradation, disinvestment from communities of color, and other material needs of BIPOC communities. Without addressing and changing the reality that the landscape of American political institutions is rooted in death and exploitation of historically oppressed populations, attempts to declare racism as a public health crisis will only perpetuate harm. The remedy could be the poison. We can avoid this by committing authentically to the following practices and actions (see Table 3).

**Table 3:** Summary of recommendations for achieving health equity through anti-racism practices.

**Address Structural Determinants of Equity** SDoE focus on structural or root causes, recognizing that addressing health disparities requires a holistic perspective. Authentic processes in mitigating inequity require addressing the unequal allocation of power and resources	**•Care over control ** Replace increased surveillance and restrictive funding with direct community partnerships. Resourcing medically underserved communities allows for self- determination of healthy futures. •Anti-racism analysis Acknowledging and reconciling that mechanisms, systems, and employees within public health institutions, medical schools, hospitals, and other health care and educational settings are rooted in racism. •Meaningful community building Integrating within existing health systems a Black and Indigenous community health approach to reducing health disparities along racialized lines. Addressing specific barriers that interfere with access to medical and public health recommendations.
**Reparations ** The case for reparations has never been clearer considering the obvious result of decades of policies leading to persistent modern day racial inequities in wealth, home ownership, and health, as examples. Avoidable racial differences in COVID-19 spread and transmission might have been decreased by up to sixty percent if reparations had previously been enacted	**•Financial accountability ** Reparations for Black descendents of enslaved persons as a solution for racial health inequities. Financial responsibility includes reparations as a solution to disrupting the seeming permanence of racism in our society. •Truth and reconciliation Healing justice is a framework that allows for analysis of power, profit, control, exploitation, ableism, oppression, violence, and trauma across health systems with a focus on addressing harm and generational healing. Centering healing justice principles and practices can serve to evaluate process and self-reflexive practice in the spirit of truth and reconciliation.
**Address Indigenous Erasure** An example of Indigenous Erasure within public health is continued genocide through data erasure and misclassification. Lack of data directly limits the ability tribal health authorities and policy makers' ability to make data-driven decisions for equitable policy and resource allocation	**•Honoring treaty rights ** The continued violation of treaty rights is a legacy of the colonial control of genocidal intent foundational to America. Decolonization is rooted in the repatriation of land and the rematriation of cultural practices and lifeways. •Decolonizing data Decolonizing data includes methods of collecting, analyzing, and interpreting data using anti-oppression and Indigenous frameworks and value systems. •Anti-blackness and indigenous erasure Addressing anti-Blackness within Indigenous communities and Indigenous erasure within Black communities needs to be addressed in dismantling racism. True accountability will not be accomplished by only addressing one without the other.
**Abolitionist Praxis ** Acknowledging the ways in which the prison industrial complex interacts and works with the medical industrial complex (MIC) is a step in changing the landscape of what is possible. Abolition requires the development of alternative solutions toward social problems; abolition is both a process and a goal.	**•BREATHE Act ** A project of the Movement for Black Lives that calls for decriminalization, elimination of police enforcement, and diversion of funding from law enforcement agencies to community-based programs that address violence and harm. •Decriminalization and decarceration From a public health and health justice critical lens, decriminalization, and decarceration are required in redefining what health, healing, and safety are without punitive measures and punishments. •Incarceration as a Public health crisis ncarceration is considered a SDoE by Healthy People 2030. Reform alone will not solve the longstanding human rights abuses and violence committed by the prison industrial complex.

### Practices to Address SDoE

We consider these three practices to be high priority in the current unique window of opportunity to advance racial equity.

#### Care Over Control

First, declarations should name the importance of prioritizing care over control (88). There were health inequities and disparities before COVID-19 that demonstrated the need to prioritize health, healing and community safety compared to prioritizing systems that oppress, surveil and harm people, especially BIPOC communities. Because of the nature of the pandemic, COVID-19 has led to the proliferation of contact tracing efforts which use mechanisms of surveillance that could further racialize and harm BIPOC, driving home the imperative that these mechanisms must be interrogated and reoriented to advance trustworthiness. Prioritizing care in this example could exemplify providing resources according to need, like personal protective equipment (PPE) and subsidized housing for essential workers who cannot physically distance. It also means being committed to the frameworks and approaches that prioritize tackling the SDoE. Resolving to shift resources toward struggling communities in order to achieve the benchmark set by those communities should be our north star.

#### Anti-racism Analysis

Public health policies must be race-explicit if they seek to achieve racial and health equity. The gaps between Black and white outcomes (89) in this nation have not improved over several decades of colorblind policy and structural racism continues to drive health inequities (90) under our current legal framework despite persistent calls for resources distributed according to need (91). By implementing evidence-based approaches, recognizing and rectifying historical injustices, we can ensure that the recovery is prescriptive and preventive, instead of reflective of the status quo.

Additionally, this anti-racist analysis needs to be applied to our disciplines to be able to work across sectors, not just in public health. Disaggregating data, utilizing race equity toolkits, and using different models are important and critical to shift where we need to go. Without acknowledging, admitting, and reconciling that our mechanisms, systems, and employees within public health institutions, medical schools, hospitals, and other health care and educational settings are rooted in racist practice themselves, we will fall short of this principle. Put another way, our current infrastructures and practices are embedded in a system that is reflective of systemic racism, bias and discrimination that we need to grapple with.

The Anti-Racism in Public Health Act of 2021 aligns with such goals and includes the creation of an anti-racism center at the CDC, embedding an anti-racist practice into COVID-19 response, and addressing police violence and brutality as a public health crisis (92). Initially introduced in 2020, this bill is being reintroduced and all states who have declared racism as a public health crisis should support this federal bill (93).

#### Meaningful Community Building

The voice of BIPOC communities, their priorities, and their self-determination and autonomy must be centered and respected in order for health institutions to achieve trustworthiness (94) and successfully end racial health inequities (95). As Critical Resistance speaks to, “Participatory community research can create capacity for us to name the problems and frame the questions” (96).

Failing to operationalize this principle will lead to harm by experts who lack critical consciousness (97) and offer solutions that do not reflect the lived reality nor address the material needs of the communities they serve. This is an example of valuing all individuals and populations evenly and equally more broadly. Partnering, developing, or integrating within existing health systems a Black and Indigenous community health approach to reducing health disparities along racialized lines. Replacing the useless label of non-compliance with descriptions of the specific barriers that interfere with one's ability to follow medical and public health recommendations (e.g., medication management support, transportation support, technology support) would be a start.

### Reparations

#### Financial Accountability

Reparations (98, 99) for Black descendants of enslaved persons in the USA is receiving attention as a solution for racial health inequities, even from mainstream medical journals (100). The case for reparations has never been clearer considering the now obvious result of decades of policies leading to persistent modern day racial inequities in wealth (101), home ownership (102), and health (90) as examples. The unjust and avoidable racial differences in COVID-19 spread and transmission might have been decreased by up to sixty percent if we had previously enacted reparations for Black descendants of persons enslaved in this country (103). Future declarations of racism as a public health crisis should certainly be financially responsible and should also openly and directly support reparations as a solution to this never-ending crisis of racism if we are serious about disrupting its seeming permanence in our society.

#### Truth and Reconciliation

It is important to address and acknowledge whether or not the populations most impacted by structural racism were harmed in the process of creating declarations. Actions that appropriate, co-opt, or censor BIPOC organizers contribute to the potential transformation of remedy to poison. As an institution rooted in structural racism, people of color operating within it often experience racialized harm, particularly when questioning white supremacy (104). Healing justice is a framework that allows for analysis of power, profit, control, exploitation, ableism, oppression, violence, and trauma across health systems (105) with a focus on addressing harm and generational healing. Although not visible within the context of this analysis for any of the declarations which is one of the strongest examples of harm, centering healing justice principles, and practices can serve to evaluate process and self-reflexive practice in the spirit of truth and reconciliation. For example in 2015, the Maine Wabanaki-Child Welfare Truth and Reconciliation Commission (TRC) released a report finding and acknowledging that the state of Maine committed a cultural genocide against its Native peoples—the Wabanaki—by forcibly removing Native children from their homes and placing them with white families (106).

### Addressing Indigenous Erasure

#### Honoring Treaty Rights

Native peoples' have inherited an audacious vision of what it means to survive an apocalypse and keep dreaming (107). At the center of this paper is an offering to truly listen and move in solidarity with the communities most impacted by racism. Compassionate activism as an antidote to non-performativity. Julian Brave NoiseCat offers that Native peoples, as well as African Americans, “know what it means to lose our world and live,” and therefore, “might have something to lend to a broader humanity that now faces its own existential crises in the form of disease and climate change.”

In 2020, a landmark Supreme Court case was passed to uphold, for the first time ever, a treaty right between a Native tribe and the US Government. *McGirt v. Oklahoma* honors that *1*9 million acres composing 47 percent of the state of Oklahoma is Native land (108, 109). The continued violation of treaty rights goes beyond the level of non-performativity and a legacy of the necropolitical colonial control of genocidal intent. This historic win acknowledged that, “at the end of the trail of tears was a promise.”

Aligned with that vision of honoring promises to the Indigenous peoples on whose land we are guests, and within the scope of public health, institutions, and governments can create meaningful health partnerships and programs with Native Health Boards, increase representation of Native and Indigenous employees, and seek to create meaningful representation within data for Native and Indigenous communities. Decolonization is not a metaphor; it is rooted in the repatriation of land and the rematriation of cultural practices and lifeways (110).

#### Decolonizing Data

At the point in the pandemic when the Navajo Nation had some of the highest COVID-19 rates in the country, the Seattle Indian Health Board received body bags instead of the requested personal protection equipment (PPE) (111). Intentional or not, this action embodies the very heart of a necropolitical dynamic in literally assigning who receives life-saving medical supplies and who receives body bags.

Decolonizing data (112), or methods of collecting, analyzing, and interpreting data using anti-oppression and Indigenous value systems (113), has implications from appropriate allocation of resources, addressing the Missing and Murdered Indigenous Women (MMIW) movement (114), to increasing funding for tribal health organizations, to decrease disparate health outcomes (115), to innovating approaches to treatment for chronic disease associated with historical trauma (116).

When the needs of those most impacted are met, it creates practices that support the broader community. Disaggregating data, utilizing culturally affirming approaches to rectify disparities, and decentralizing health programming by funding existing community-based responses all begin with a decolonized approach to health research and data. Decolonizing data (117) recognizes that research is an indigenous practice and integral part of wisdom that created and sustained the abundance (118) that existed prior to first contact.

#### Addressing the Relationship Between Anti-blackness and Indigenous Erasure

To meaningfully move forward in addressing racism as a public health crisis, it is important to name and discuss lateral violence between communities of color. White supremacy is predicated on the public dehumanization of the Black body and the erasure and silencing of Indigenous voices (119). Addressing anti-Blackness within Indigenous communities, and Indigenous erasure within Black communities is a deep and ongoing internal accountability process that also needs to be addressed in dismantling racism. True accountability will not be accomplished by only addressing one without the other. As put by Malinda Maynor Lowery (Lumbee) in response to an incident on anti-Blackness in the Native community, “What do Lumbees lose when Black lives matter? Nothing except our colonized minds” (120). This includes complicating narratives between Black and White (121), and recognizing (122) that Black Americans are from an Indigenous African diaspora (123).

### Abolition

As defined by Critical Resistance, “abolition is both a practical organizing tool and a long-term goal” (124). Prison industrial complex (PIC) abolition, is focused on eliminating punitive measures, practices, policing and policies that control, surveil, and harm people. Acknowledging the ways in which the prison industrial complex interacts and works with the medical industrial complex (MIC) in these declarations will help reimagine what is possible by taking steps to move toward other models of health, healing and safety. Changing the landscape of what is possible, abolition requires the development of alternative solutions toward social problems (125). Radical legislation, like the BREATHE Act, takes steps toward abolition through a reimagining of public safety, community care, and infrastructure. As public health professionals, we have a responsibility, “to apply a comprehensive, holistic approach to prevent and proactively address the trauma, repression and disruption of communities” (126, 127). The following are examples of policies and ideologies that inform an abolitionist public health practice.

#### BREATHE Act

The BREATHE Act, a project of the Movement for Black Lives, calls for decriminalization, elimination of police enforcement (in activities including but not limited to drug use and possession, sex work, loitering, sleeping in public, minor traffic violations), and diversion of funding from law enforcement agencies to community-based programs that address violence and harm. These activities are within the context of ensuring they do not criminalize communities, including mental health intervention, violence prevention and intervention, and conflict mediation programs, particularly in the communities currently most affected by police harassment and violence (128).

With a divest/invest focus, the BREATHE Act focuses on opportunities to invest in “new, non-punitive, non-carceral approaches to community safety” while divesting from heavy policing and carceral methods in order to build healthy, sustainable and equitable communities. The BREATHE Act is an example of how to move the needle forward from a policy standpoint in reimagining what the world could be.

Furthermore, as posed by Critical Resistance and other public health entities such as the American Public Health Association, “community-centered strategies for addressing harm and violence have the potential to increase public safety without the violence associated with policing,” citing that decriminalization and decarceration are a means to achieve and move toward health equity by investing in community-based solutions and models to drive and move toward healing and reconciliation processes and strategies (128).

For example, in subsection 3, BREATHE Act specifically acknowledges and centers the importance of an approach that is beyond “high-quality, equitable, and accessible healthcare.” By creating opportunities to “incentivize state and local government to make health equity-focused policy changes,” it opens access to ensuring health equity through use of dollars and goods for food, economic and health access. Put another way, as the subsection illustrates, “ensuring health equity, including equity for Black, Latinx, AAPI, Indigenous, LGBT, low-income, homeless, disabled, and undocumented individuals…” (129) through a competitive grant is a way to begin to abolish current infrastructures and begin to create new ways of innovation and bring abundance to communities that are often left scarce.

From other aspects of BREATHE Act, it is very clear that the underlying legislative description and research are situated in creating policies and practices that build capacity for public health by involving more community voices to drive the conversations from the upstream, rather than not being transparent and leaving the door open for more troubles and issues down the line. While it may not be perfect in that a national policy packet may need to be modified and changed for the local and state context in the US, these declarations have an opportunity to utilize similar context, language, and foresight as the BREATHE Act to build power and create space for health equity conversations, innovations and practices for the future.

Abolition and policies, like the BREATHE Act, centering practices and policies that take on decarceration and decriminalize, would drive the momentum further in what these declarations and resolutions could mean for the future of health in the US and beyond. With investments in community practices, avenues for training and skill-building as well as succession planning with community members, public health advocates and practitioners and healers, models of care and safety can be codified and created as well as evaluated and assessed over time for their effectiveness and sustainability.

#### Decriminalization and Decarceration

Decarceration is the process of reducing the number of people who are currently incarcerated and diverting those who might become embedded into the prison industrial complex (130). Decriminalization is the act of removing criminal sanctions against an act, article or behavior (131). When it comes to public health, health justice, decriminalization, and decarceration, efforts are a way to center abolition because it requires redefining what health, healing, and safety are without punitive measures and punishments. Like COVID-19 and other public health emergencies and crises, prisons, and jails pose a great community risk not only for those who are directly impacted, but for the construction of how safety and care is determined in a community. Policing and surveillance are also a large part of our lives. This rapid acceleration of technologies, of data, of biometrics is sounding the alarm. Big Data, from “the abstraction of the catastrophic loss of human life, and the necessary torture required to maintain plantations, needed to serve the owners who were removed from the daily abuse of the literal rows and fields” with slavery to the systems we are all too familiar with today “used to control, surveil, and enact violence to maintain power structures and ensure profit,” scientific oppression, aggressive public policy, and harmful algorithmic design need to be stopped (132). Health systems are now capable of using algorithms that use predictive analysis to determine care in hospitals, creating a new web of harm that the MIC is a part of.

These algorithms, like many in education and policing, can discriminate, as Dr. Ruha Benjamin states as “the New Jim Code” (133). Therefore, with these declarations, there has be a conscious effort to recognize how to work with communities to ensure that there is a balance between transparency, interoperability, and freedom of privacy.

#### Incarceration as a Public Health Crisis

With an increasing focus on fighting the prison industrial complex, ongoing concerns about surveillance and control over care, and COVID-19 spread, public health workers have stepped up to support decarceration and decriminalization but have not supported abolition at a larger scale. The US has the highest rate of incarceration in the world (134) with an incarcerated population that has increased by 700 percent since 1970 (135). Over 60 percent of incarcerated persons (136) are people of color. Importantly, prisons and jails (137) are often amplifiers of infectious diseases so it is not surprising that they reveal the highest rates of COVID-19 transmission (138) which is unjust and completely preventable. While the momentum across sectors and groups has been evident for many decades (96), even as incarceration is being considered a SDoE by Healthy People 2020 and 2030, it is increasingly clear that reform of the prison industrial complex will not solve the longstanding human rights abuses and violence that incarcerated persons and those impacted by the prison industrial complex deal with. More public health workers should take a clear stance in support of abolition. Because public health implications are supported by clear evidence of the inadequacies of carceral reform, advancing steps toward abolition, by centering formerly incarcerated people, those impacted by the prison industrial complex, abolitionist organizers, and organizations around the world, is also critical.

## Conclusion

As we work to dismantle deeply embedded systems of oppression and correct historical injustices that have plagued the Americas for hundreds of years, public health practitioners, healers and community members will have to continue to be critical and thoughtful about how to reimagine the world. For those most impacted, an infrastructure that prioritizes and maximizes opportunities for profit from oppression must be abolished. Our bodies, our people, deserve better than to become numbers, figures or models. It is time for public health to reckon with its history, how it is currently operating, and think more radically and critically of how to move away from non-performativity toward health equity. Healing justice, disability justice, and intersectional frameworks that consider how interlocking systems of oppression operate, are integral to this process. Without recognition that non-performative declarations will by default support the maintenance of a white supremacist society, there is no space for the conversations necessary to drive generative change in undoing structural racism.

Desmond Tutu said in an interview about forgiveness, to forgive but to never forget (139). We believe we can remedy the poisons of the past in solidarity with truth, reconciliation, and justice. As Dr. William C. Jenkins states, “researchers, practitioners, and community members will all need to build on the field's history of fighting racism and its health effects if health disparities are to be eliminated” (140). Therefore, by declaring racism as a public health crisis, we're naming what so many scholars, pioneers, and communities have known is a part of a larger structural, root cause that will prevent us from a more just and liberated future. CDC has recently declared racism as a public health threat, again illuminating that the shift to move us all forward is here and it's time. Considering too, an ever-evolving digital frontier and oppressive systems interlocked, as we tried to demonstrate throughout this paper, the possibility of amplifying the medical apartheid and unjust systems that do not prioritize care and community is terrifying and insidious. As we move toward rapid automation, robots and roads with automatic cars, telemedicine and big data are being used to “optimize” the world in a way we have only imagined in popular culture. However, can we ideally say technology and science with its legacies in harm, oppression and overt destruction won't be used in the same way in a “possible” future?

“Sola dosis facit venenum” means that the dose makes the poison. This basic principle of toxicology points to the need for titration for this reason.Therefore, he field of public health, medicine and arguably, science, must reconcile and commit to making meaningful change to address structural racism and deepening health disparities. It has to rexamine the ways in which “evidence” and “data” is collected, what building with communities and people will require to create new models of care and safety, and what we have to learn together to drive that change. Therefore, we believe accountability is that titration process.

Accountability is a process of integrity and responsibility that requires health professionals to move shoulder to shoulder with the communities that have been most impacted. Instead of being rooted in punishment, revenge or superficiality, accountability should be anchored on the values of growth, transformation, healing, and liberation (53). For the declarations to be able to move the needle, there has be accountability and recognition of futures that we have made invisible and blueprints that the past laid out that we never quite finished, such as the Ten Point Plan by the Black Panther Party.

Without meaningful accountability (6) (e.g., realistic budget and timelines, thoughtful-decision making, community inclusion, appropriate frameworks), policies, such as declaring racism a public health crisis, could become weaponized as pharmakon as officials and leaders seeking cover for rising unrest and dissatisfaction with progress toward racial justice and equity. This means that longitudinal studies on health policies and implemented public health practices, through legal epidemiology and cross-sector analyses (141), with communities leading and creating the solutions with invested, sustainable resources is key. For example, policies have to be studied in a variety of ways, such as through mechanism studies that examine how laws affect environments, behaviors and health over the long and short term (141). It'll require examining cultural and socio-political theories that are race-conscious, clear on decentralized models of care and prioritizing that communities know and are the ones who are most equipped to drive what places, people and resources are needed to solve the problems they're most acquainted with. For example, as the language of intersectionality grows in popular media, researchers have to be critical and thoughtful about their work. As COVID-19 has demonstrated, and as scholars have noted, it has been communities who have once again been the most knowledgeable and actionable analyses to center and support themselves and each other (142). Put another way, it has not been traditional public health agencies or surveillance systems, but community-based organizations and policy think tanks, that have the analyses of the disproportionate and structural impacts of the pandemic on people (142). The more that public health and health infrastructures evaluate and change their current model of care and safety, the more we can envision a praxis that is more situated closer to putting people before profit or performance.

It'll also mean abolishing structures, institutions and places that are not serving the purpose of the people, that only seek to extract and profit off communities. It's because there are also futures that we can envision and imagine without policing, without destruction, and without harm so that when we conjure names, like Octavia Butler and Dr. Ruha Benjamin, we have to remember that those futures exist too.

### Limitations

Due to small sample size of declarations, and the lack of available data, there is a need for more rigorous policy study in this area. Additionally, this paper focuses on racism within the context of the United States, but there remains imperative need to address racism from a global perspective, particularly as it relates to the impact of US imperialism. There is a need to go beyond what currently exists. To truly dismantle white supremacy and structures of anti-Blackness and colonialism, there is a need for a new grammar of futurity (143) in health and social policy.

## Author's Note

The authors of the paper are members of the Equal Health Campaign Against Racism (CAR); Racism as a Public Health Crisis Taskforce. CAR is a global grassroots network of health care professionals, organizers, and activists committed to dismantling racism within health care. In its first year, 250 active members and 1,500 health professionals and allies engaged through various CAR actions. Members have launched 23 chapters across 10 countries. CAR came into being in 2017, under call from past president of the American Public Health Association, CJ and leadership from MM, founder of Equal Health. LP is a public health advocate, ESTJ that flexes from Sensing to Thinking and Judgement, an elderly millennial, born and raised in the Midwest. LP contributions to the field of public health include starting the movement to declare racism a public health crisis. As a multiracial Indigenous person in diaspora, PR (Mēxihcatl) approaches anti-racism and decolonization work as an act of ancestor honoring. AE Medical Director at CityBlock Health, and a leader in national and international medical education with demonstrated leadership in hospital management as a previous Chief Medical Officer and Intensive Care Unit Director in Haiti. CA is a holistic scientist and health educator born and raised in the American South. A black womanist/feminist, scientist and a solarpunk enthusiast, Courtni is an afrofuturist scholar, healer in progress, data geek who sees how the world can be carved and forged into a place where healing, health and tech can be in harmony. Her praxis is at the intersection of critical race theory, intersectionality, and scientific discovery, hoping to center and continue to learn more in disability, healing, and data justice. LL research and organizing are centered in abolition, decolonial feminisms, and transnational solidarity. AC is a community organizer in Catalonia, Spain and was instrumental in supporting the passage of the first international declaration of racism as a public health crisis, in solidarity with undocumented youth organizers.

## Author Contributions

All authors contributed to the conceptualization, research, writing, and editing of this paper.

## Conflict of Interest

CA works as a consultant for the CDC.

The remaining authors declare that the research was conducted in the absence of any commercial or financial relationships that could be construed as a potential conflict of interest.
